# Applying New Research Criteria for Diagnosis of Early Alzheimer's Disease: Sex and Intelligence Matter

**DOI:** 10.4061/2009/638145

**Published:** 2009-08-12

**Authors:** U. Beinhoff, H. Tumani, M. W. Riepe

**Affiliations:** ^1^Department of Psychiatry, Mental Health and Old Age Psychiatry, Charité Medical University, 14050 Berlin, Germany; ^2^Department of Neurology, University of Ulm, 89081 Ulm, Germany; ^3^Division of Mental Health & Old Age Psychiatry, University of Ulm, Ludwig-Heilmeyer Strasse 2, Ulm, 89312 Günzburg, Germany

## Abstract

Alzheimer's disease (AD) can be diagnosed according to new research criteria proposed recently (Dubois et al., 2007). Diagnosis is made on grounds of episodic memory deficits and one pathological biomarker: cerebrospinal fluid (CSF) or structural/functional imaging. Goal was to investigate the dependence of episodic memory function on material (verbal, visuospatial), gender and premorbid intellectual ability (IQ). The new research criteria of AD were applied retrospectively using data of 68 patients (Mini-Mental-Status Examination, MMSE ≥ 22) from a university memory clinic. Women with lower IQ performed worse on visuospatial episodic memory than women with higher IQ and men with the same IQ. Thus, women with lower IQ appear to be particularly vulnerable to *visuospatial* episodic memory deficits despite similar CSF tau values indicating a similar activity of the neurodegenerative process. Gender, premorbid IQ, and visuospatial material need to be considered in the assessment of episodic memory breakdown applying the newly proposed research criteria for the diagnosis of AD.

## 1. Introduction

Alzheimer's disease (AD) is one of the most common diseases of old age [1–3]. Established research criteria for clinical diagnosis of Alzheimer's disease (AD) require diagnosis of dementia syndrome and exclusion of possible causes of dementia other than AD [4]. Recently, revised research criteria for positive diagnosis of Alzheimer's disease—with or without clinical dementia syndrome—were proposed [5]. Other than the previous set of criteria diagnosis is not performed by exclusion; the new set of criteria aim for a positive diagnosis of AD. The revised criteria demand a significant episodic memory impairment which does not improve with cuing plus an abnormal result for at least one biomarker: mediotemporal atrophy on structural neuroimaging, an AD-typical pattern on functional neuroimaging (PET or SPECT), or AD-typical results for Abeta- and tau-protein in cerebrospinal fluid (CSF) analysis. With this framework, diagnosis of AD does not require presence of dementia syndrome.

The correlation between severity of dementia and neuropathology is loose [6–8]. One likely cause is that the disease is driven by diffusible oligomeric amyloid assemblies causing synaptic failure rather than by the amyloid depositions alone [9, 10]. The clinical presentation of failure of synaptic transmission in affected individuals, however, varies with premorbid variables [11]. Epidemiologic evidence suggests that individuals with higher IQ [12], or higher educational or occupational attainment [13, 14], or participation in leisure activities [15] have an increased capacity for buffering the burden of disease. Not only the capacity against decline may differ according to these variables but also the *pattern* of decline [16] which in addition may be gender-dependent [17–19]. 

Investigations on *healthy* controls suggest a female advantage in episodic memory with predominantly verbal material and a male advantage for visuospatial tasks [20–30]. The impact of gender on memory in *aging*, however, further is modified by general intellectual abilities [31].

It was the goal of the present study to analyze the influence of both, gender and general intellectual abilities on verbal and visuospatial episodic memory performance in patients diagnosed with newly revised research criteria that require at least one positive biomarker evidence for securing AD diagnosis [5].

## 2. Methods

The study was done in accordance with the ethical standards of the University Clinics (Berlin and Ulm). All persons gave their informed consent prior to their inclusion in the study. 

### 2.1. Study Sample

From the cohort of patients assessed in a specialized dementia outpatient university hospital service 68 patients with mild cognitive deficits (Mini-Mental-Status Examination, MMSE  ≥ 22) were selected fulfilling either the diagnosis of Alzheimer's Disease according to NINCDS-ADRDA criteria and DSM-IV or the criteria for Mild Cognitive Impairment as defined by Petersen [32]. Patients were only selected that contained necessary data (IQ, neuropsychology, CSF (tau-protein, Abeta-protein), or cerebral imaging) to apply the revised research criteria for probable Alzheimer's disease [5]. For final analysis patients were included when they had an episodic memory deficit that does not significantly improve with cuing plus at least one supportive finding (either abnormal CSF tau or Abeta concentrations or medial temporal lobe (MTL) volume loss assessed by a qualitative rating using MRI). Subjects with other neurological or psychiatric diseases that could explain memory deficits were excluded, as well as subjects who received medication that could interfere with cognitive function. Basic characteristics of this sample are described in Table 1.

### 2.2. Neuropsychological Tests

**General Intellectual Abilities**

It has been shown that intelligence (i.e., reading ability) predicts incident dementia better than education measured in number of years [33]. As a measure of crystallized (general) intelligence we used a German vocabulary test (Wortschatztest, WST) [34]. Raw scores from the WST were converted into IQ scores, according to the manual. Patients were grouped according to general intellectual ability (low general intellectual ability with IQ  < 100 and high intellectual ability with IQ  > 100).

**Dementia Severity**

Dementia severity was staged with the MMSE [35]. In the present study, we included subjects with a MMSE score  ≥ 22 because our goal was to describe gender differences in the early stages of AD and patients with more advanced stages of the disease are rarely able to complete the comprehensive neuropsychological tests applied in this study.

**Episodic Memory**

Verbal episodic memory with the immediate and delayed recall of a word list was tested with the German version of the California Verbal Learning Test (CVLT) as previously applied [17, 36]. The word list is a shopping list whose items can be grouped into four categories (fruits, drinks, tools, clothes). This allows a cued recall, where subjects have to recall all items belonging to a specific category. As a measure of episodic memory (for immediate and delayed recall), the percent rate of correctly recalled words was used. Immediate and delayed recall of geometric forms was tested with the visual reproduction test (Wechsler Memory Scale, WMS) [37]. The percent rate of correctly recalled shapes is reported. In accordance with the Petersen criteria [32], the cut-off was set at 1.5 SD.

### 2.3. Analysis of Cerebrospinal Fluid

Cerebrospinal fluid (CSF) routine parameters were analyzed. CSF for analysis of tau-protein was frozen immediately after lumbar puncture and stored at −80°C. CSF tau concentrations were determined at the University of Ulm using a sandwich ELISA (INNOTEST hTAU, Innogenetics) as described [38], which recognizes normally phosphorylated and unphosphorylated tau. The assay was adapted according to the protocol supplied with the kit (laboratory reference ranges: <200 pg/mL and <300 pg/mL for control individuals below 65 and older than 65 years, resp.), and tau concentrations of the samples were estimated from standard curves made for each assay. Tau levels >200 pg/mL (below 65 years) and >300 pg/mL (older than 65 years) were regarded as indicative of a neurodegenerative process. The analytical sensitivity of the assay was 75 pg/mL, and the intraassay and interassay variation was <8%. CSF Abeta-amyloid 1–42 levels were determined using a sandwich ELISA developed several years ago [39] and now commercially available (Innogenetics, Zwijndrecht, Belgium). The assay was performed according to the protocol supplied with the kit, and CSF Abeta concentrations of the samples were estimated from standard curves made for each assay. Abeta protein levels below 550 pg/mL were regarded as indicative of a neurodegenerative process.

### 2.4. Structural Neuroimaging

Structural cerebral imaging with magnetic resonance imaging (MRI) was classified by *visual rating* as representing normal findings or increased medio-temporal atrophy. A detailed quantitative regional analysis was beyond the scope of the present study.

### 2.5. Statistical Analysis

Four groups were compared in this study: male and female AD patients with either high or low intellectual ability. In order to reduce alpha-error probability, statistical tests were applied in a hierarchical manner, that is, differences between 2 single groups were only investigated if the overall analysis of all four groups revealed significant differences. In order to provide comparability to other studies, data are presented as mean values with standard deviation (SD) and ranges. All statistical analyses were carried out using nonparametric testing (chi-square, Mann-Whitney-U-Test, Kruskal-Wallis test) applying the statistics program SPSS (SPSS 15.0 for Windows, Chicago, Ill, USA).

## 3. Results

The four groups were equal according to number of patients (N, Chi-square, *P* > .05) and age (Kruskal-Wallis-Test, *P* > .05) but differed in their MMSE scores (Kruskal-Wallis test, *P* = .03). Men in the high IQ group had higher MMSE scores than men in the low IQ group (U-Test, *P* = .028) and women in the high IQ group (U-Test, *P* = .044).

The number of abnormal *biomarkers* (CSF tau, CSF Abeta, MRI) was not significantly different between the four groups (Kruskal-Wallis test, *P* > .05). CSF biomarkers revealed similar levels for CSF Abeta-protein in all four groups (Kruskal-Wallis test, *P* > .05) but differences in CSF tau levels (Kruskal-Wallis test, *P* = .039). Women in the high IQ group had higher CSF tau values than men in the high IQ group (U-Test, *P* = .012). Although women with higher IQ had numerically increased values for CSF tau than women with lower IQ, this difference reached no significance. CSF tau values were similar for women and men in low IQ patients.

The results of the *episodic memory* function for verbal and visuospatial material are presented in Table 2. Overall analysis revealed similar results for all four groups in episodic memory function for *verbal* material (Kruskal-Wallis test, *P* > .05).

However, episodic memory significantly differed between the four groups for *visuospatial* material (Kruskal-Wallice test, immediate recall: *P* = .018; delayed recall: *P* = .001). In delayed recall male AD patients achieved better test results than female patients (low IQ group: U-Test, *P* = .009; high IQ group: U-Test, *P* = .036). High IQ male patients showed better results than low IQ male patients in immediate recall (U-Test, *P* = .03). In delayed recall, women with higher IQ performed better than women with lower IQ (U-Test, *P* = .026).

## 4. Discussion

Recently, research criteria for the diagnosis of Alzheimer's disease (AD) have been proposed [5]. With this framework, diagnosis of AD is achieved on finding an episodic memory deficit plus AD-typical biological markers (e.g., CSF tau protein and Abeta-protein, atrophy on structural imaging). Considering the gender-specific profile of memory in AD patients [17] and modulation of memory in healthy adults by general intellectual abilities [40, 41], it was the goal to explore both, the influence of intelligence and gender on verbal and visuospatial memory in AD patients.

It has been proposed that CSF t-tau concentrations reflect the intensity or *activity* of the neurodegenerative process [42, 43]. This is supported by the respective CSF t-tau concentrations in various diseases with different progression rates (CJD  > AD  > ALS  > MS). In addition, within a single disease entity, the CSF t-tau concentration likely indicates a faster progression rate [44–46]. In AD, the tau levels in CSF correlate with the annual atrophy rate [47]. Abeta on the other hand has been proposed to give a measure of *how far* the disease process already has proceeded [42]. This is supported by the findings that the degree of atrophy measured on structural cerebral imaging [47] or Abeta deposition evaluated in vivo with positron emission tomography [48] correlate with CSF Abeta levels.

### 4.1. The Role of IQ

**Figure 1:**
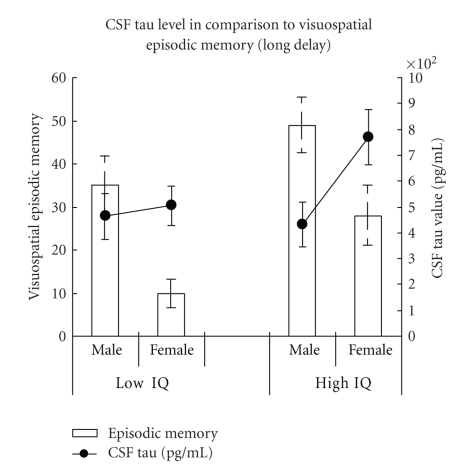
Visuospatial episodic memory performance (bars indicate % of maximum score) in comparison to CSF tau values (lines) in male and female AD patients. Higher memory scores indicate better performance. Higher CSF tau values indicate more advanced disease (activity).

High functioning elders are particularly difficult to diagnose, as they score too high on standard tests for which the norms were established in populations of average general intellectual abilities [49]. Comparable to other studies in healthy adults [12, 40, 41], we found significant differences in cognitive function in AD patients depending on their premorbid IQ level. Male AD patients with higher IQ had better test results regarding their immediate memory for visuospatial material than male AD patients with lower IQ. CSF tau values (i.e., disease activity) were not different for male AD patients. These findings indicate a higher capacity for men with higher intelligence. Similar results were found in female patients for long delayed recall of visuospatial material. Although their CSF tau value was numerically (but not significantly) increased, women with higher IQ showed even better test results on their visuospatial episodic memory than women with lower IQ (see Figure 1), also indicating a higher capacity for women with higher intelligence.

### 4.2. The Role of Gender

In the present study, we found gender-differences in episodic memory function for visuospatial material depending on IQ level. In the *lower IQ group*, men performed better than women. CSF tau (i.e., disease activity) values were similar in both groups. This result indicates that despite same levels of disease activity, male AD patients are better in remembering visuospatial material despite same levels of disease activity compared to female AD patients. In the *higher IQ group*, women also performed worse than men. CSF tau values for the high IQ group were increased for women, showing that disease activity was higher in this group. In a further analysis controlling for disease activity (CSF tau-value), gender differences in episodic memory function disappear, that is, no advantage for males was observed. We speculate that women with higher intellectual abilities might use strategies (e.g., verbalization of the material) which may explain the absence of gender differences for visuospatial material at least in the high IQ group. For future studies, material which is less subject to verbalization than even the present geometric patterns should be used.

Gender differences in the verbal domain that are well established for *healthy* controls [31] were not observed in the present study on patients with mild AD. Most likely, aging [28] and the disease process obscures gender specificities observed in healthy adult individuals.

## 5. Conclusion

Our results show that both IQ and gender modulate episodic memory in AD patients in a modality specific fashion (verbal versus visuospatial). Women with lower IQ appear to be particularly vulnerable to visuospatial episodic memory deficits. The dependence on gender and general intellectual capacity in episodic memory breakdown needs to be considered in the newly proposed research criteria for the diagnosis of early Alzheimer's disease.

## Figures and Tables

**Table 1:** *Characterization of the study sample: ranges, mean, and standard deviations in brackets*. MMSE: Mini-Mental Status Examination; WST: German Vocabulary Test (Wortschatztest) as a measure of Intelligence; CSF: cerebrospinal fluid; Low IQ (IQ < 100); High IQ (IQ > 100).

	Low IQ (*N* = 35)		High IQ (*N* = 33)	
	Male	Female	Male	Female
*N*	20	15	18	15
Age	57–80	58–85	56–81	58–85
	(68.0 ± 7.0)	(71.1 ± 8.0)	(70.0 ± 8.6)	(68.7 ± 8.6)
MMSE	22–30	22–29	24–30	22–30
	(26.3 ± 2.3)	(25.7 ± 2.2)	(27.8 ± 1.7)*	(26.1 ± 2.4)
WST	17–30	12–30	31–39	31–39
	(23.8 ± 4.0)	(24.5 ± 4.9)	(34.4 ± 2.5)	(33.7 ± 2.1)
CSF tau	100–1658	74–1242	75–1428	200–1500
	(463 ± 387)	(494 ± 298)	(431 ± 366)	(770 ± 413)**^§^**
CSF Abeta	109–1140	262–1380	100–1351	221–1153
	(624 ± 310)	(523 ± 287)	(700 ± 377)	(533 ± 275)
Number of Abnormal biomarkers^#^	One: *N* = 10	One: *N* = 4	One: *N* = 10	One: *N* = 4
	Two: *N* = 8	Two: *N* = 11	Two: *N* = 6	Two: *N* = 10
	Three: *N* = 2	Three: *N* = 0	Three: *N* = 2	Three: *N* = 1

*: significant difference to high IQ females and low IQ males; ^§^: significant difference to high IQ males; ^#^: CSF tau, CSF Abeta, MRI.

**Table 2:** Values for episodic memory function (in % of maximum score): means (SD).

Immediate recall	Verbal		Visuospatial	
	Low IQ	High IQ	Low IQ	High IQ

Male	44.7 (18.1)	55.6 (14.4)	52.2 (18.8)^$^	65.0 (15.3)
Female	40.4 (21.9)	52.9 (24.6)	46.0 (15.5)	55.6 (17.1)

Long delayed recall	Verbal		Visuospatial	
	Low IQ	High IQ	Low IQ	High IQ

Male	24.4 (18.9)	36.8 (21.7)	34.6 (30.4)	48.8 (27.5)
Female	19.2 (27.3)	30.8 (27.4)	10.3 (14.8)*	27.9 (26.2)^§^

*: significant difference to high IQ females and low IQ males; ^§^: significant difference to high IQ males; ^$^: significant difference to high IQ males.
